# Protostane and Fusidane Triterpenes: A Mini-Review

**DOI:** 10.3390/molecules18044054

**Published:** 2013-04-05

**Authors:** Ming Zhao, Tanja Gödecke, Jordan Gunn, Jin-Ao Duan, Chun-Tao Che

**Affiliations:** 1 Department of Medicinal Chemistry & Pharmacognosy, and WHO Collaborative Center for Traditional Medicine, College of Pharmacy, University of Illinois at Chicago, Chicago, IL 60612, USA; 2 Jiangsu Key Laboratory for TCM Formulae Research, Nanjing University of Traditional Chinese Medicine, Nanjing 210046, China

**Keywords:** *Alisma*, alisol, fusidane, protostane, triterpene

## Abstract

Protostane triterpenes belong to a group of tetracyclic triterpene that exhibit unique structural characteristics. Their natural distribution is primarily limited to the genus *Alisma* of the Alismataceae family, but they have also been occasionally found in other plant genera such as *Lobelia*, *Garcinia*, and *Leucas*. To date, there are 59 known protostane structures. Many of them have been reported to possess biological properties such as improving lipotropism, hepatoprotection, anti-viral activity against hepatitis B and HIV-I virus, anti-cancer activity, as well as reversal of multidrug resistance in cancer cells. On the other hand, fusidanes are fungal products characterized by 29-*nor* protostane structures. They possess antibiotic properties against staphylococci, including the methicillin-resistant *Staphylococcus aureus* (MRSA). Fusidic acid is a representative member which has found clinical applications. This review covers plant sources of the protostanes, their structure elucidation, characteristic structural and spectral properties, as well as biological activities. The fungal sources, structural features, biological activities of fusidanes are also covered in this review. Additionally, the biogenesis of these two types of triterpenes is discussed and a refined pathway is proposed.

## 1. Introduction

**Figure 1:**
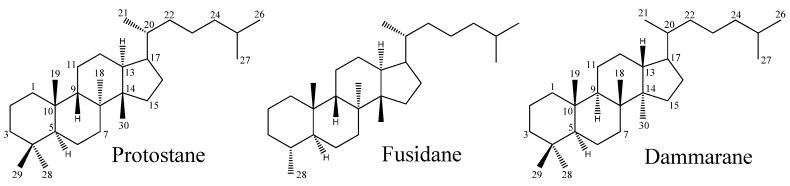
Skeletons of the protostane, fusidane, and dammarane triterpenes.

Protostane triterpene (PT) is a stereoisomer of the tetracyclic triterpene dammarane, displaying characteristic stereostructures in positions 8*α*-CH_3_, 9*β*-H, 13*α*-H, and 14*β*-CH_3_ (Figure 1). As early as in 1969, Hattori and associates first reported the isolation of 3*β*-hydroxy-4*β*-methylfuside-17(20), [16,21-cis],24-diene and 3*β*-hydroxy-4*β*-methylfuside-13(17),24-diene from a culture broth of the fungus *Cephalosporium caeruleus* [1]. Considered to be the “prototype” of steroids, the skeleton of these isolates was named “protostane”. Accordingly, two structures representing the first examples of the protostane class are 3*β*-hydroxyprotosta-17(20)*Z*, 24-diene, and 3*β*-hydroxyprotosta-13(17), 24-diene, respectively. 

To date, a total of 59 PTs are reported from higher plants, but no glycosidic derivatives have ever been found. The majority of PTs were isolated from plants belonging to the genus *Alisma* (family Alismataceae). Thus, they are the major and most characteristic components of the Chinese medicine Alismatis Rhizoma, the dried rhizome of *Alisma orientale* (Sam.) Juzep. or *A.* *plantago-aquatica* L. [2]. A number of* in vitro* and* in vivo* biological activities have been associated with PTs. They include lipotropic and hepatoprotective activities, anti-viral properties against hepatitis B virus and HIV-I, anti-tumor activity, anti-complement activity, and reversal of multi-drug resistance in cancer cells. For instance, alisol A 24-acetate exhibited marked anti-cholesterolemic effects in an* in vivo* assay (blood cholesterol levels in hyper-cholesterolmic rats were reduced by 61%) [3]; 13*β*,17*β*-epoxyalisol A, and 16-oxoalisol A inhibited 100% and 60% of D-galactosamine-induced damage in liver cells, respectively [4]; alisol F 24-acetate showed promising effects against hepatitis B virus infections by inhibiting HBsAg (HBV surface antigen) and HBeAg (HBV e antigen) with IC_50_ values of 7.7 *μ*M and 5.1 μM, respectively [5].

Fusidane triterpene (FT) belongs to another small group of tetracyclic *nor*-triterpenes, which can be structurally considered as 29-*nor* protostane triterpenes (Figure 1). To date, 18 naturally occurring FTs have been reported. Among them, fusidic acid has been used in the clinic as an antibiotic for decades; and it remains a unique and promising agent due to the significant potencies against staphylococci, especially the methicillin-resistant *Staphylococcus aureus* (MRSA). Fusidic acid has relatively low toxicity; it is non-allergic and has little cross-resistance with other clinically used antibiotics.

We herein present the first comprehensive review on these two groups of triterpenes. This paper deals with their natural occurrence, isolation and structure elucidation, structural and spectral characteristics, biological activities, as well as a proposed biogenetic pathway. 

## 2. Protostane Triterpenes

### 2.1. Distribution of Protostane Triterpenes in Higher Plants

Of the 59 PTs reported from higher plants (Table 1), most were isolated from *Alisma* (Alismataceae), in particular, *A. orientale*. They are therefore considered to be of chemotaxonomical significance for the *Alisma* genus. In a few reports, PTs have also been found in three other species, namely, *Lobelia chinensis* Lour. (Lobeliaceae) [6], *Garcinia speciosa* Wall. (Guttiferae) [7], and *Leucas cephalotes* (Roth) Spreng. (Labiatae) [8].

**Table 1:** Naturally occurring protostane triterpens.

No.	Name	M.F.	Source	Sub-group ^a^	Bioassays conducted ^b^	References
**1**	Alisol A	C_30_H_50_O_5_	*Alisma orientale*,*A. planta-aquatica*	I	5	[3,9,10,11,12,13,14]
**2**	Alisol A 24-acetate	C_32_H_52_O_6_	*A. orientale*,*A. planta-aquatica*	I	7	[3,5,9,10,11,12,15,16]
**3**	Alisol F	C_30_H_48_O_5_	*A. gramineum*,*A. orientale*,*Lobelia chinensis*	I	2	[5,6,11,17,18,19]
**4**	Alisol G (25-anhydroalisol A)	C_30_H_48_O_4_	*A. orientale*,*A. planta-aquatica*	I	4	[5,10,11,13,19]
**5**	13*β*,17*β*-Epoxyalisol A	C_30_H_50_O_6_	*A. orientale*	I	2	[4,5,19]
**6**	11-Deoxyalisol A	C_30_H_50_O_4_	*A. orientale*	I	-	[20]
**7**	11-Deoxy-13*β*,17*β*-epoxyalisol A	C_30_H_50_O_5_	*A. orientale*	I	-	[20]
**8**	25-O-Methylalisol A	C_31_H_52_O_5_	*A. orientale*	I	-	[20]
**9**	16-Oxoalisol A	C_30_H_48_O_6_	*A. orientale*	I	1	[4,20]
**10**	16-Oxo-11-anhydroalisol A	C_30_H_46_O_5_	*A. orientale*	I	-	[21]
**11**	16-Oxo-23-deoxyalisol A	C_30_H_48_O_5_	*A. orientale*	I	-	[21]
**12**	Alizexol A(alisol F 24-acetate)	C_32_H_50_O_6_	*A. orientale*	I	1	[5,6,22]
**13**	Alisol H	C_30_H_46_O_4_	*A. orientale*	I	1	[11,23]
**14**	Alismaketone B 23-acetate	C_32_H_50_O_6_	*A. orientale*	I	-	[11]
**15**	25-Anhydroalisol A 11-acetate	C_32_H_50_O_5_	*A. orientale*	I	-	[24]
**16**	25-Anhydroalisol A 24-acetate	C_32_H_50_O_5_	*A. orientale*	I	-	[24]
**17**	Neoalisol	C_30_H_46_O_4_	*A. orientale*	I	-	[24]
**18**	13*β*,17*β*-Epoxyalisol A 24-acetate	C_32_H_52_O_7_	*A. orientale*	I	-	[25]
**19**	Alisol O	C_32_H_48_O_5_	*A. orientale*	I	-	[5]
**20**	24-Deacetylalisol O(11-anhydroalisol F)	C_30_H_46_O_4_	*A. orientale*	I	-	[26]
**21**	25-Anhydroalisol F	C_30_H_46_O_4_	*A. orientale*	I	-	[27]
**22**	11,25-Anhydroalisol F	C_30_H_44_O_3_	*A. orientale*	I	-	[28]
**23**	Alisol X	C_30_H_46_O_3_	*A. orientale*	I	-	[29]
**24**	Alisol B	C_30_H_48_O_4_	*A. orientale*	II	8	[9,11,12,14,15,17,30,31]
**25**	Alisol B 23-acetate	C_32_H_50_O_5_	*A. orientale*,*A. planta-aquatica*	II	12	[3,5,9,11,14,15,16,17,32,33]
**26**	11-Deoxyalisol C	C_30_H_46_O_4_	*A. gramineum*,*A. orientale*,*A. planta-aquatica*	II	-	[18,23,34]
**27**	Alisol D(13*β*,17*β*-epoxyalisol B 23-acetate)	C_32_H_50_O_6_	*A. orientale*,*A. planta-aquatica*	II	-	[21,34]
**28**	16*β*-Hydroxyalisol B 23-acetate	C_32_H_50_O_6_	*A. gramineum*,*A. orientale*,*A. planta-aquatica*	II	1	[18,20,31,35]
**29**	16*β*-Methoxyalisol B 23-acetate	C_33_H_52_O_6_	*A. gramineum*,*A. planta-aquatica*	II	-	[18,35]
**30**	11-Deoxyalisol B	C_30_H_48_O_3_	*A. orientale*	II	2	[11,31,36]
**31**	11-Deoxyalisol B 23-acetate	C_32_H_50_O_4_	*A. orientale*	II	-	[36]
**32**	Alisol C	C_30_H_46_O_5_	*A. orientale*	II	-	[20]
**33**	Alisol C 23-acetate	C_32_H_48_O_6_	*A. orientale*	II	5	[3,14,15,20,31,33]
**34**	11-Deoxyalisol C 23-acetate	C_32_H_48_O_5_	*A. orientale*	II	-	[20]
**35**	16*β*,23*β*-Oxidoalisol B	C_30_H_46_O_4_	*A. gramineum*,*A. orientale*	II	-	[18,20]
**36**	13*β*,17*β*-Epoxyalisol B	C_30_H_48_O_5_	*A. orientale*	II	-	[20]
**37**	11-Deoxy-13*β*,17*β*-epoxyalisol B 23-acetate	C_32_H_50_O_5_	*A. orientale*	II	-	[20]
**38**	Alismaketone A 23-acetate	C_32_H_50_O_6_	*A. orientale*	II	1	[37]
**39**	Alisol I	C_30_H_46_O_3_	*A. orientale*	II	1	[11,23]
**40**	Alisol J 23-acetate	C_32_H_46_O_6_	*A. orientale*	II	-	[23]
**41**	Alisol K 23-acetate	C_32_H_46_O_6_	*A. orientale*	II	1	[11,23]
**42**	Alisol L 23-acetate	C_32_H_46_O_5_	*A. orientale*	II	1	[11,23]
**43**	Alisol M 23-acetate	C_32_H_48_O_7_	*A. orientale*	II	1	[11,23]
**44**	Alisol N 23-acetate	C_32_H_50_O_6_	*A. orientale*	II	1	[11,23]
**45**	Alisol Q 23-acetate	C_32_H_48_O_6_	*A. orientale*	II	-	[38]
**46**	Alisol E (epi-alisol A)	C_30_H_50_O_5_	*A. orientale*	III	1	[9,11,37]
**47**	Alisol E 23-acetate	C_32_H_52_O_6_	*A. orientale*	III	1	[11,19]
**48**	Alisol E 24-acetate	C_32_H_52_O_6_	*A. orientale*	III	-	[25]
**49**	Alismalactone 23-acetate	C_32_H_48_O_7_	*A. orientale*	IV	2	[11,37]
**50**	3-Methyl alismalactone 23-acetate	C_33_H_50_O_7_	*A. orientale*	IV	1	[11]
**51**	Alisol P	C_30_H_48_O_7_	*A. orientale*	IV	-	[39]
**52**	Alismaketone C 23-acetate	C_32_H_48_O_6_	A. orientale	IV	1	[11]
**53**	Alisolide	C_26_H_36_O_4_	*A. orientale*	V	-	[39]
**54**	3-Oxo-13*β*,23-dihydroxy-24,24-dimethyl-26,27-dinorprotost-13(17)-en-25-oic acid	C_30_H_48_O_5_	*A. orientale*	VI	-	[39]
**55**	Garciosaterpene A	C_32_H_50_O_4_	*Garcinia speciosa*		1	[7]
**56**	Garciosaterpene B	C_30_H_48_O_3_	*G. speciosa*		-	[7]
**57**	Garciosaterpene C	C_30_H_46_O_3_	*G. speciosa*		1	[7]
**58**	Leucastrin A	C_31_H_54_O_2_	*Leucas cephalotes*		-	[8]
**59**	Leucastrin B	C_30_H_52_O_3_	*L. cephalotes*		-	[8]

^a^ More information is described in 2.2.1; ^b^ The number of bioassay in which the compound has been tested.

### 2.2. Protostane Triterpenes from Alisma

#### 2.2.1. Characteristic Structural Features and Classification

PTs mainly differ in their prevalent oxygenation pattern at the positions C-2, 3, 11, 13, 16, 17, 23, 24 and 25 (Figure 1). A keto group is always found at C-3, with the only exception of alismaketone A 23-acetate (**38**). Keto groups may also be present at C-2, C-11, C-16 or C-23. Acetylation often occurs at positions C-23 or C-24, and rarely at C-11; and epoxy group may be present at positions 24(25), 13(17), or 16(17). In some PT derivatives, an oxygen bridge may form between C-17/C-23, C-16/C-23, or C-16/C-24, giving rise to a five-, six-, or seven-membered oxygen-bridged ring systems. On the other hand, most PTs possess a double bond at the 13(17) position, and in some, a double bond also occurs at positions 11(12), 12(13), or 25(26). The hydroxyl and epoxy groups are present in *β*-orientation, with only a few exceptions such as alisols J 23-acetate (**40**), E (**46**), E 23-acetate (**47**), and E 24-acetate (**48**).

*Alisma* PTs can be conveniently divided into the following six structural sub-groups (see Table 1 for sub-group assignment):

(I) Alisol A series: (20*R*, 23*S*, 24*R*) configuration; without a 24,25-epoxy group;
(II) Alisol B series: bearing a 24,25-epoxy group;
(III) Alisol E series: (20*R*, 23*S*, 24*S*) configuration; without a 24,25-epoxy group;
(IV) *Seco*-PTs: 2,3-*seco* and 13,17-*seco* derivatives (the affix *seco* is used to denote the cleavage of a ring in a parent structure);
(V) *Nor*-PTs: 24,25,26,27-tetra-*nor*-protostane (The affix *nor* is used to denote the elimination of one or more carbons from the parent structure);
(VI) Rearranged PTs.

#### 2.2.2. Spectral Characteristics in IR, UV, MS, and NMR

##### 2.2.2.1. IR, UV, and MS Spectra

A typical IR spectrum of the PT structure is characterized by the presence of five types of absorption bands, *viz*. hydroxyl (3400–3500 cm^−1^), ester carbonyl (indicative of acetylation, 1720–1740 cm^−1^), unconjugated carbonyl (1705–1745 cm^−1^), *α*,*β*-unsaturated carbonyl (1660–1700 cm^−1^), and olefinic group (around 1630 cm^−1^). 

The typical UV spectrum of a PT may display absorption maxima at 243–246 nm and around 285 nm. These bands are indicative of enone functionality and dienone group, respectively. 

General speaking, the molecular ion of PT can readily be observed by EI-MS. When soft ionization techniques such as ESI-MS and FAB-MS are used, the mass spectra will display the corresponding quasi-molecular ions, [M+H]^+^, [M+Na]^+^, or [M−H]^−^. In Q-TOF-MS, the protonated molecular ion [M+H]^+^ is readily detectable, and collision-induced dissociation tandem mass spectrometry (CID-MS-MS) can produce characteristic fragments resulting from the dissociation of the bond between C-23 and C-24, which is useful for differentiation of isomers containing an acetyl unit on the C-23 or C-24 positions [40]. 

##### 2.2.2.2. Nuclear Magnetic Resonance (NMR) Spectra

NMR has proved to be the most powerful tool for structural elucidation of organic compounds. As a matter of fact, most PT structures were elucidated primarily through interpretation of their NMR spectroscopic data. NMR analysis also provides an additional tool for stereochemical determination.

In the ^13^C-NMR spectra of *Alisma* PTs, the keto carbon (C-3) resonance is consistently found around *δ_C_* 220. When present, the keto carbonyl carbon at C-11 [conjugated with the 12(13) double bond], C-16 [conjugated with the 13(17) double bond], and C-23 can be observed around *δ_C_* 199, 208, and 212, respectively. The carbon signals of 13(17) double bond are often found in the neighborhood of *δ_C_* 135 (C-17) and 137 (C-13); yet their chemical shifts may move downfield to *δ_C_* 137–140 (C-17) and 176–179 (C-13) if conjugation with a keto group at C-16 occurs. When a conjugated system is present between the double bonds at positions 11(12) and 13(17) and a keto group at C-16, the chemical shift values of these carbons are observable around *δ_C_* 121 (C-11), 138 (C-12), 171 (C-13), 137 (C-17) and 207 (C-16). For the same conjugation system, but in the absence of the keto group at C-16, the chemical shift values of the conjugated double bonds are usually found around *δ_C_* 121 (C-11), 130 (C-12), 138 (C-13) and 134 (C-17). The resonances of the terminal 25(26) double bond, when present, can be observed around *δ_C_* 144 (C-25) and 114 (C-26). In comparison with the PT structures of alisol A series, alisol B series of compounds reveal upfield chemical shifts for the C-24 (*δ_C_* 65–68), C-25 (*δ_C_* 58), and C-26 (*δ_C_* 19). They represent shifts of about10, 14, and 7 ppm, respectively. 

In the ^1^H-NMR spectra (typically acquired in CDCl_3_), distinctive signals can be observed for the methyl groups in the neighborhood of *δ_H_* 0.8–1.5. In addition, the presence of two broad singlets around *δ_H_* 4.93 and 4.97 are indicative of an olefinic CH_2_ group [the terminal double bond present at the 25(26) position].

#### 2.2.3. Alisol A Series

**Figure 2:**
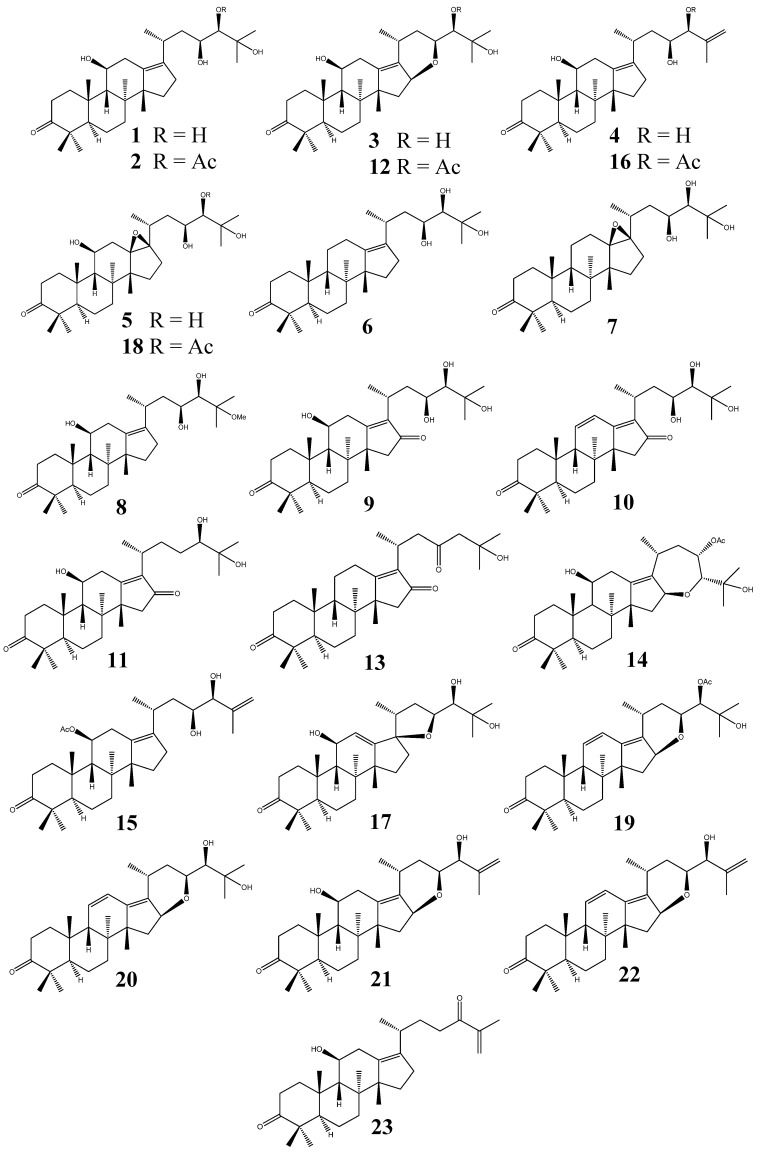
Structures of the alisol A series of protostanes.

The alisol A series (Figure 2) is characterized by the (20*R*, 23*S*, 24*R*) absolute configuration and the absence of a 24,25-epoxy group. Alisol A (**1**) and alisol A 24-acetate (**2**) were the first PTs isolated from the rhizome of *Alisma orientale* in 1968 [9]. Both planar and stereochemical structures of **1** were established by chemical derivatization and an X-ray crystallographic analysis of its (23,24)-acetonide 11-monobromoacetate derivative [41]. 

On the other hand, the structure of **2** was initially erroneously identified, but was later revised [17]. Alisol F (**3**), alisol G (25-anhydroalisol A, **4**), and 13*β*,17*β*-epoxyalisol A (**5**) were also isolated from *A. orientale* [19]. The *β*-oriented 16,23-epoxy structure of alisol F (**3**) was verified by NOESY. The absolute configuration at C-24 was elucidated as *R* by the application of modified Mosher’s method [19]. Later, alisol F (**3**) was also isolated from the rhizome of *A. gramineum* [18]. Alisol G (**4**) was determined to be the anhydro-derivative of **1**, verified by chemical conversion [19]. **5** was obtained as a new naturally occurring compound, while it had been previously prepared from **1** by epoxidation with *m*-chloroperbenzoic acid [19]. 

Alizexol A (alisol F 24-acetate, **12**) was isolated in 1995 [22], but it was not until 2001 that the C_24_-*R* absolute configuration was determined using a chemical correlation method [42]. A unique structure bearing a C_23_-keto group, alisol H (**13**) [23], as well as alismaketone B 23-acetate (**14**) [11], possessing a seven-membered 16,24-epoxy ring system, were also isolated from the rhizome of *A. orientale*. The epoxy-ring connection between C-16 and C-24 was confirmed by HMBC correlations observed between 16-H and C-24. The stereochemistry of **14** was determined to be the same as that of alisol A (**1**) by chemical correlation,* i.e.*, reduction of **14** with Li in ethylenediamine resulted in dihydroalisol A [11].

25-Anhydroalisol A 11-acetate (**15**) [24] is the only example of a PT structure that possesses an acetate group at the C-11 position; all other PTs bear acetates on C-23 or C-24, if present. 25-Anhydroalisol A 24-acetate (**16**) was identified as a new naturally occurring product [24], but it had been previously reported as an anodic oxidation product of alisol A (**1**) [43]. 

Alisol O (**19**) was identified as 24(*R*)-hydroxyprotosta-11,13-diene 24-acetate 16(*S*),23(*S*)-epoxide based on interpretation of NMR spectra [5]. The 24-deacetyl derivative (24-deacetylalisol O/11-anhydro-alisol F, **20**) were independently obtained by two research groups; but it was perplexing that two opposite optical rotation values were reported for the same structure: ${\left[\alpha \right]}_{D}^{20}$ = −39.64 (*c* 0.05, MeOH) [26] and ${\left[\alpha \right]}_{D}^{20}$ = +10.2 (*c* 0.4, MeOH) [28].

Two derivatives of alisol F (**3**), namely 25-anhydroalisol F (**21**) and 11,25-anhydroalisol F (**22**), were isolated from the rhizome of *A. orientale* [27,28]. Alisol X (**23**) is a unique PT possessing a C_24_-keto group [29]. 

Other PTs belonging to the alisol A series include 11-deoxyalisol A (**6**), 11-deoxy-13*β*,17*β*-epoxyalisol A (**7**), 25-*O*-methylalisol A (**8**), 16-oxoalisol A (**9**), 16-oxo-11-anhydroalisol A (**10**), 16-oxo-23-deoxyalisol A (**11**), neoalisol (**17**) and 13*β*,17*β*-epoxyalisol A 24-acetate (**18**) [20,21,24,25]. 

Additionally, the isolation of alisol A (**1**), alisol A 24-acetate (**2**), and alisol G (**4**) have been reported from the rhizome of *A. planta-aquatica* [10].

#### 2.2.4. Alisol B Series

**Figure 3:**
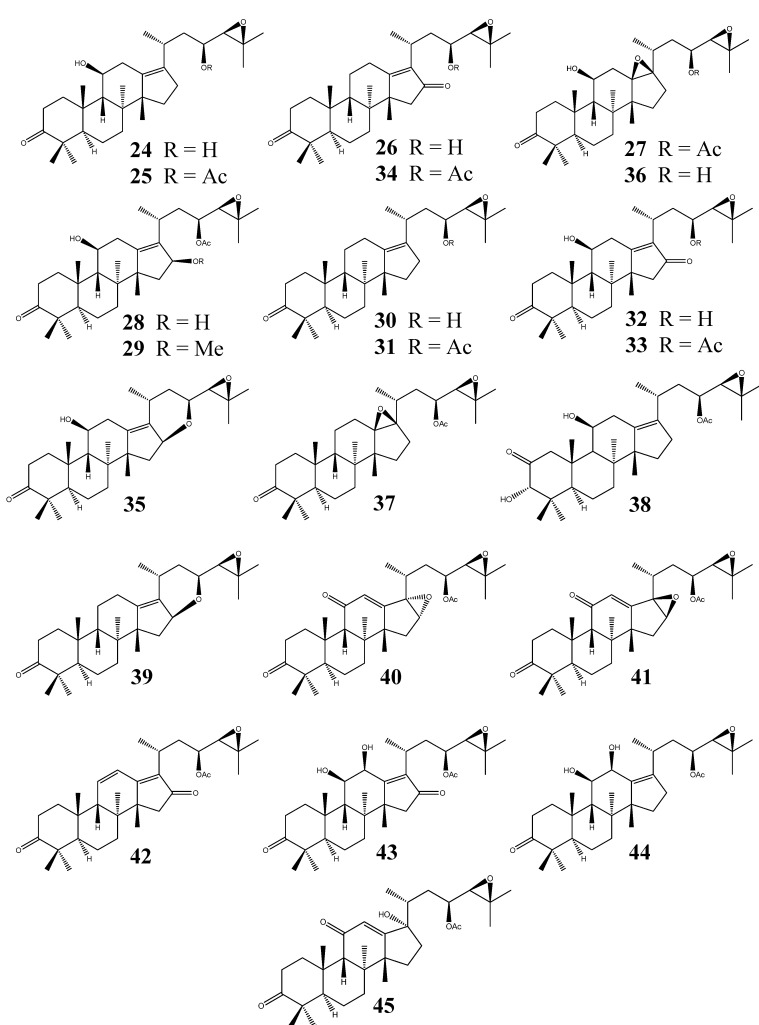
Structures of the alisol B series of protostanes.

In contrast to the alisol A series, alisol B PTs (Figure 3) possess a 24,25-epoxy group in their structure. Alisol B (**24**) and alisol B 23-acetate (**25**) were isolated from the rhizome of *A. orientale* in 1968, but their structures were later revised [9,17]. An X-ray crystallographic analysis of **25 **was published in 2003 [44]; however, the stereochemistry was erroneously assigned as 5*β*-H, 8*β*-CH_3_, 9*α*-H, 13*β*-H, and 14*α*-CH_3_, which is opposite to the configurations of other PTs. 

11-Deoxyalisol C (**26**) and alisol D (13*β*,17*β*-epoxyalisol B 23-acetate) (**27**) were isolated from *A. planta-aquatica* [34]. The former compound possesses a 13(17)-double bond and a 16-keto group whereas the latter contains a 13,17-epoxy group. When alisol B 23-acetate (**25**) was treated with *m*-chloroperbenzoic acid, alisol D (**27**) was obtained, presumably due to acid attack at the 13(17) double bond from the hindered *β*-side. The presence of a 13*β*,17*β*-epoxy group in the structure of **27** was further confirmed by X-ray crystallographic analysis [45]. 

The structure elucidation of 16*β*-hydroxyalisol B 23-acetate (**28**) and 16*β*-methoxyalisol B 23-acetate (**29**) from *A. planta-aquatica* were primarily based on NMR spectroscopic data. The 11-OH was assigned to the *β*-configuration due to a large coupling constant for 11-H (*J* = 11.1 and 10.0 Hz). The *β*-configuration of the methoxy or hydroxy group at the C-16 position was determined based on NOE enhancement of the 16-H and 11-H signals upon irradiation of the 28-CH_3_ [35].

11-Deoxyalisol B (**30**) and its 23-acetate derivative (**31**) were isolated from the fresh rhizome of *A. orientale* in 1993 [36]. This was the first investigation on fresh *Alisma* plant materials. 

Alisol C (**32**) and its 23-acetate derivative (**33**), 11-deoxyalisol C 23-acetate (**34**), 16,23-oxidoalisol B (**35**), 13*β*,17*β*-epoxyalisol B (**36**), and 11-deoxy-13*β*,17*β*-epoxyalisol B 23-acetate (**37**) were all obtained from the dried rhizome of *A. orientale* [20]. The X-ray crystallographic analysis of **33** revealed an unusual boat/boat configuration of the A and B rings in the solid state [21].

Alismaketone A 23-acetate (**38**) possesses a keto group and a hydroxy group at positions C-2 and C-3, respectively. This is in contrast with most PTs previously isolated from *Alisma* plants, all of which bear a keto group only at C-3. The 2-keto-3-ol structure of **38** was elucidated based on detailed examination of its COSY and HMBC spectra. The absolute configuration at C-3 was determined to be *S* based on the result of modified Mosher’s method [37].

Phytochemical investigation of the dried rhizome of *A. orientale* further led to the isolation of alisols I (**39**), J 23-acetate (**40**), K 23-acetate (**41**), L 23-acetate (**42**), M 23-acetate (**43**), and N 23-acetate (**44**) [23]. Alisol I (**39**) was found to contain a six-membered 16,23-epoxy ring and a three-membered 24,25-epoxy ring. Compounds **40** and **41 **were identified as two stereoisomers bearing 16*α*,17*α*-epoxy and 16*β*,17*β*-epoxy ring systems, respectively. Compounds **43** and **44** represent the only two known protostane derivatives that contain a 11*β*,12*β*-dihydroxy group. Alisol Q (**45**) was isolated from the rhizomes of *A. orientale*, which was a unique PT bearing a 17-OH in the structure [38]. Additionally, **26**, **28**, **29**, and **35** have also been reported from the rhizome of *A. gramineum* [18].

#### 2.2.5. Alisol E Series

**Figure 4:**
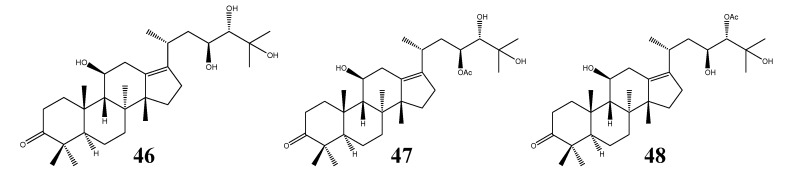
Structures of the alisol E series of protostanes.

Protostanes of the alisol E series (Figure 4) possess an absolute configuration of 24*S*. Alisol E (epi-alisol A, **46**) was first isolated from the rhizome of *A. orientale* in 1968 and later identified as a C_24_-*S* epimer of alisol A (**1**) [9,37]. 

It was the first example of a C_24_-*S* PT isolated from natural source. Detailed NMR spectroscopic data were not reported until 1993 in the study of alisol E 23-acetate (**47**) as the second C_24_-*S* protostane [19]. The stereochemistry at C-24 was confirmed by applying the modified Mosher’s method. In addition to chemical methods, NMR data provided further evidence for the differentiation between the *R* and *S* configuration at C-24 in these molecules [8,39]. The isolation of alisol E 24-acetate (**48**) was reported in 2002 [25].

#### 2.2.6. *Seco*-Protostane Triterpenes

**Figure 5:**
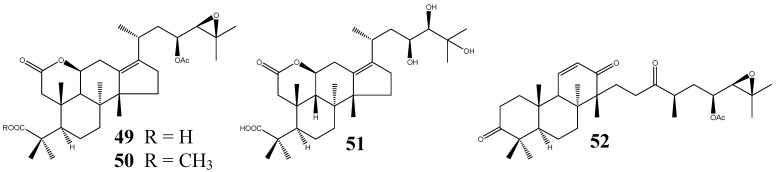
Structures of the *seco*-protostanes.

To date, only four *seco*-PTs have been found (Figure 5), all from the dried rhizome of *A. orientale*. Three of them are 2,3-*seco*-protostanes, namely alismalactone 23-acetate (**49**), 3-methyl alismalactone 23-acetate (**50**), and alisol P (**51**). The 2,3-*seco*-protostane structure of **49** was elucidated on the basis of NMR spectroscopic data and chemical correlation with alisol B 23-acetate (**25**) [37].

Compound **50** was first reported as a methylation product of **49 **and later purified from the plant material [11,37]. Alisol P (**51**) differs from **49** by possessing a 23,24,25-trihydroxy side-chain. Its stereochemistry was assigned to be 20*R*,23*S*,24*R* by NOESY experiment as well as by comparing the NMR data with those of alisol A (20*R*,23*S*,24*R*, **1**) and alisol E (20*R*,23*S*,24*S*, **46**) [39]. The other *seco*-derivative is a 13,17-*seco*-protostane, alismaketone C 23-acetate (**52**). Its absolute stereochemistry was determined by chemical correlation with alisol B 23-acetate (**25**) by O_3_ oxidation to cleave the olefinic group at 13(17). Subsequent treatment with SOCl_2_ in pyridine yielded **52** [11]. 

#### 2.2.7. *Nor*-Protostane Triterpenes and Rearranged Protostane Triterpenes

**Figure 6:**
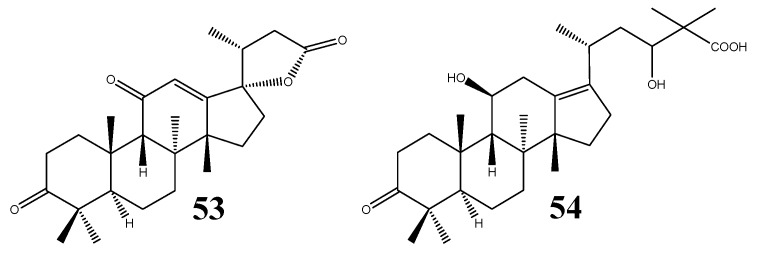
Structures of the *nor*- and rearranged protostanes.

To date, two structures of *nor*- and rearranged protostanes are known (Figure 6);* i.e.*, alisolide (**53**) and 3-oxo-11*β*,23-dihydroxy-24,24-dimethyl-26,27-dinorprotost-13(17)-en-25-oic acid (**54**) [39]. 

The former compound was confirmed as a 24,25,26,27-tetra-*nor*-protostane, for which the C_20_-*R* stereochemistry was proposed based on biogenetic evidence, while the C_17_-*S* configuration was implied by the NOESY results, in particular the correlation observed between 12-H and 21-CH_3_ [39]. Prior to the isolation of **53**, 24,25,26,27-tetra-*nor*-PT structures were considered chemical products of oxidative reactions of PTs [1,46]. Compound **54** was isolated from *A. orientale* and alisol B (**24**) was proposed to be its biosynthetic precursor [39].

### 2.3. Protostane Triterpenes from Other Plant Species

**Figure 7:**
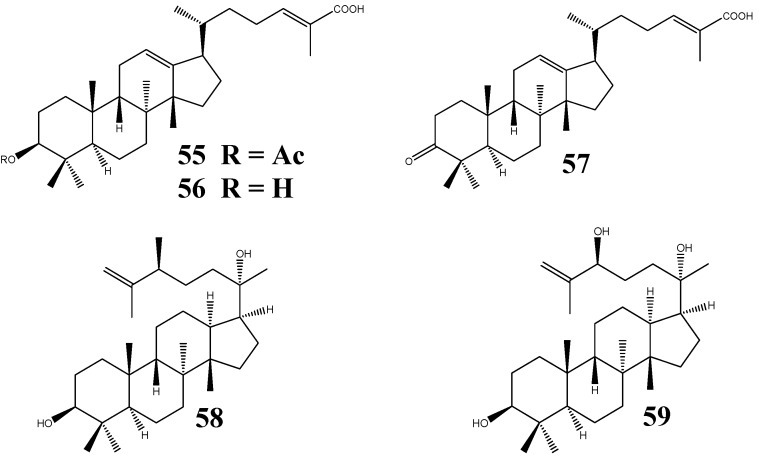
Structures of protostanes from plants outside *Alisma* genus.

Three new protostanes, garciosaterpenes A (**55**), B (**56**), and C (**57**) (Figure 7), were isolated from the trunk bark and stem of *Garcinia speciosa* Wall. (Guttiferae). Their chemical structures were proposed to be 3-acetoxy-protosta-12,24-diene-26-oic acid, 3-hydroxy-protosta-12,24-diene-26-oic acid, and 3-keto-protosta-12,24-diene-26-oic acid, respectively, based on NMR data and chemical reactions. Compounds **55** and **57** displayed inhibitory activities against HIV-1 reverse transcriptase and in the syncytium assay [7]. 

From the whole herb of *Leucas cephalotes* Spreng. (Labiatae), two PTs named leucastrins A (**58**) and B (**59**) (Figure 7) were obtained [8]. They were identified to be (3*S*,17*S*,20*S*,24*S*)-3,20-dihydroxy-24-methyl-protost-25-ene and (3*S*,17*S*,20*S*,24*S*)-3,20,24-tihydroxy-protost-25-ene, respectively. The former compound possesses an unusual 24-methyl group. DIFNOE results suggested that the A ring of both structures is in a chair conformation, with axial 10-CH_3 _group and equatorial 3-OH orientations. The B and C rings are in boat and chair conformation, respectively, with both 8-CH_3_ and 14-CH_3_ groups in axial orientation. The junctions of rings A, B, C, and D were all found to be in a *trans* conformation. Alizexol A (alisol F 24-acetate, **12**) was also isolated from the dried herb of *Lobelia chinensis* Lour. (Lobeliaceae) and its structure was confirmed by X-ray crystallographic analysis [6].

### 2.4. Biogenesis of Protostanes

**Scheme 1:**
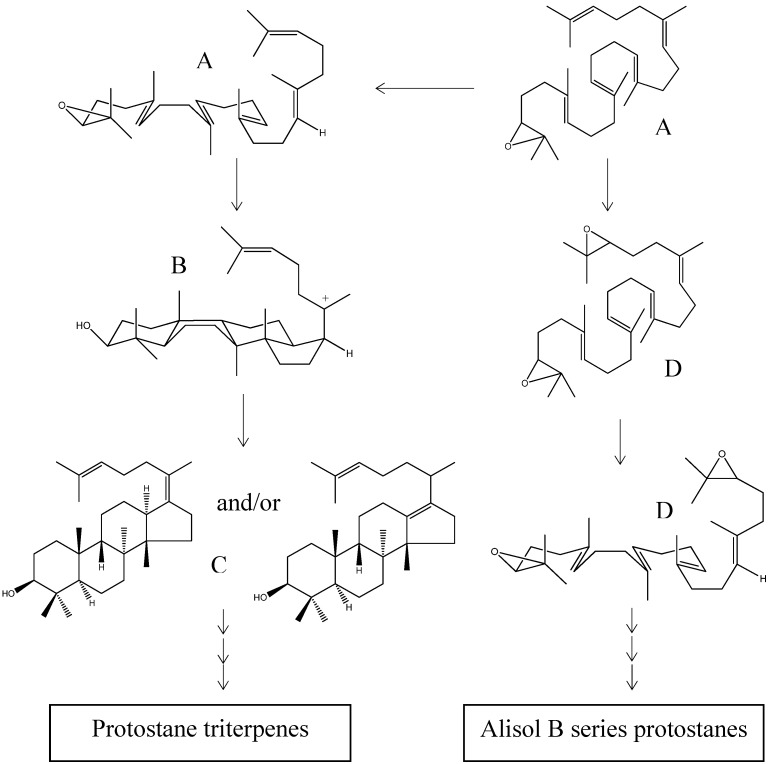
Proposed biogenetic sequence of protostanes. A: 2,3-(*S*)-oxidosqualene; B: protosteryl C-20 cation; C: protosterols; D: 2,3-(*S*)-22,23-(*S*)-bis-oxidosqualene

In higher plants, 2,3-(*S*)-oxidosqualene is generally considered to be the biosynthetic precursor of triterpenes and phytosterols through a cascade of cyclizations and rearrangements. Squalene epoxidase is responsible for the conversion of 2,3-(*S*)-oxidosqualene to 2,3-(*S*)-22,23-(*S*)-bis-oxidosqualene prior to the cyclization steps [47]. At the start of triterpene synthesis, 2,3-(*S*)-oxidosqualene (Scheme 1; structure A) adopts a pre-organized *chair*-*boat*-*chair* conformation, followed by the protonation of the epoxy ring, which triggers a cascade of ring-forming reactions resulting in a 6.6.6.5-fused tetracyclic protosteryl C-20 cation with a 17*β*-side chain (Scheme 1; structure B). Under the control of the specific “protosterol synthase”, this cation is directly deprotonated, either without rearrangement or with a 17*α*-hydride shift, to yield protosterol (Scheme 1; structure C), which is presumed to be the initial intermediate in the biosynthesis of protostane skeleton.

**Scheme 2:**
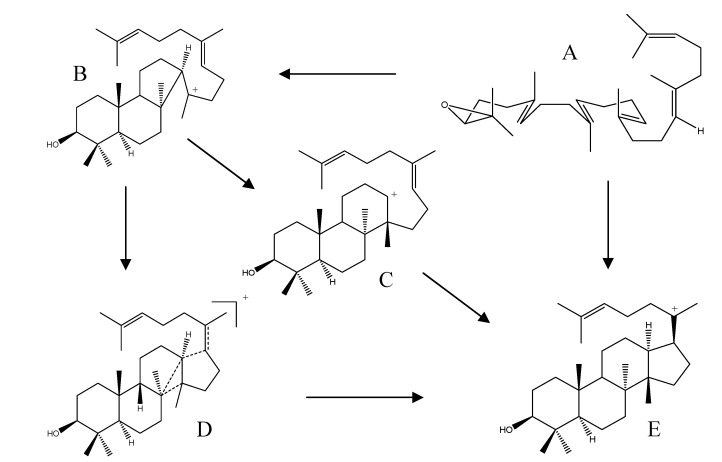
Proposed mechanism of protosteryl C-20 cation formation. A: 2,3-(*S*)-oxidosqualene; B: Markovnikov cation; C: anti-Markovnikov cation; D: transition state structure; E: protosteryl C-20 cation.

The formation of the protosteryl C-20 cation is a key step in the synthesis pathway and is also an important intermediate in the biosyntheses of phytosterols and steroidal triterpenes. The cyclization of oxidosqualene (Scheme 2; structure A) to yield the protosteryl C-20 cation (Scheme 2; structure E) was initially considered to be a concerted reaction,* i.e.*, a non-stop process without passing through stabilized intermediates. Due to a growing body of experimental and theoretical evidence, it is now largely accepted that the cyclization first yields a tricyclic Markovnikov tertiary cation possessing a five-membered C-ring (Scheme 2; structure B). However, how the six-membered C-ring and five-membered D-ring are formed remains controversial. Corey* et al.* suggested that the Markovnikov cation undergoes a ring expansion to a 6.6.6-fused tricyclic anti-Markovnikov secondary cation (Scheme 2; structure C). This hypothesis is supported by theoretical evidence provided by Jenson and Jorgensen. On the other hand, Hess proposed that the Markovnikov cation is the first intermediate in the cascade reactions, the expansion of the C-ring is a concerted reaction, and the formation of the five-membered D-ring is accomplished via a transition state structure (Scheme 2; structure D) [48,49,50,51,52]. 

**Scheme 3:**
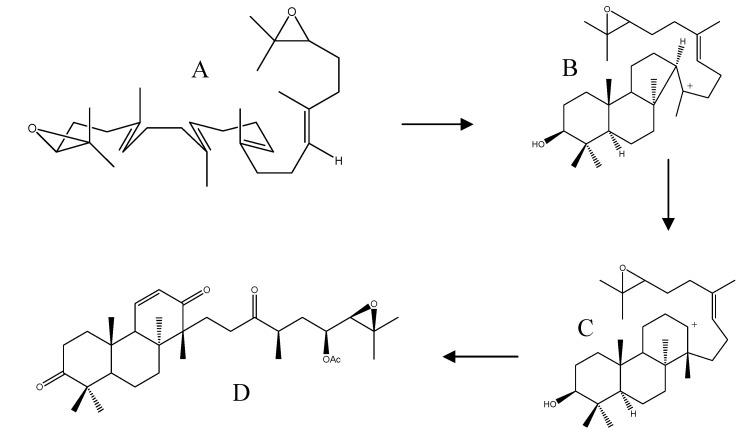
Proposed biosynthesis of alismaketone C 23-aceate (**52**). A: 2,3-(*S*)-22,23-(*S*)-bis-oxidosqualene; B: 24,25-epoxy markovnikov cation; C: 24,25-anti-epoxy markovnikov cation; D: alisomaketone C 23-acetate (**52**).

The isolation of alismaketone C 23-acetate (**52**) from *A. orientale* seemed to have provided a key piece of evidence to support the Corey hypothesis. Thus, the biosynthesis was presumed to originate from a 24,25-epoxy anti-Markovnikov cation (Scheme 3, structure C) by direct elimination of a proton, followed by oxidative structure modifications (Scheme 3). 

In the fresh rhizomes of *Alisma* plants, protostanes belonging to the alisol B series have been demonstrated to be the major components [53,54]. It was speculated that these 24,25-epoxides turn into compounds of the alisol A series during the drying process of the rhizomes, thus leading to the increase in the amounts of the latter in dried rhizome samples.

It is now proposed that the biogenesis of *Alisma* PTs starts with the 2,3-(*S*)-22,23-(*S*)-bis-oxidosqualene pathway (Scheme 1; structure D). This is in accordance with the reported biosynthesis of 24,25-epoxycholesterol (which is processed in a shunt of the mevalonate pathway, as a parallel pathway to cholesterol synthesis) [55,56]. Squalene monoxoygenase has been reported to catalyze the downstream reactions leading to 2,3-(*S*)-22,23-(*S*)-bis-oxidosqualene, which subsequently undergoes a number of transformation steps which are catalyzed by a yet unidentified enzyme complex, but via the known 24,25-epoxy protosterol.

### 2.5. Biological Activities of Protostane Triterpenes

#### 2.5.1. Lipotropic and Liver-Protective Activity

Compounds belonging to the alisol A group **1**, and **2** and the alisol B derivatives **25** and **33** were found to display marked anticholesterolemic effects. Inclusion of 0.1% of either of these compounds in the diet for hypercholesterolemic rats would reduce the cholesterol levels by more than 50%, compound **2** being most potent resulting in 61% reduction [3]. In addition, **2**, **25** and **33** were able to protect mice against CCl_4_-induced liver damage, as indicated by a modulation of serum glutamine-pyruvic transaminase and triglyceride levels, with **33** being the most effective protectant [33]. Moreover, alisol A derivatives **5 **and **9** were found to inhibit 100% and 60% of D-galactosamine-induced liver damage* in vitro*, respectively [4]. 

#### 2.5.2. Anti-viral Activity

##### 2.5.2.1. Activity against Hepatitis B Virus (HBV)

The alisol A derivatives **2**, **3**, **4**, **5**, **12**, and the alisol B type **25** were reported to exhibit protective activity against hepatitis B viral infections [5]. Compound **12** showed the most promising effects by inhibiting HBsAg (HBV surface antigen) and HBeAg (HBV e antigen) with IC_50_ of 7.7 μM and 5.1 μM, respectively, while cytotoxic effects were only observed at much higher concentrations [50% cytotoxicity concentration (CC50) = 142.7 μM].

A number of synthetic alisol A derivatives have been prepared and evaluated for their* in vitro*anti-HBV activity and cytotoxicity in a structure-activity relationship study [57,58,59]. The results suggested that acylation of the hydroxy groups at positions 11, 23, and 24 decreased the cytotoxicity. It was concluded that the carbonyl function at C-3 might is of importance for the activity. On the contrary, the 25(26) double bond of alisol A analogues might be crucial for the anti-HBV activity [57,58,59].

##### 2.5.2.2. Anti-HIV-I Activity

Two PT derivatives isolated from plants outside the *Alisma* genus, **55** and **57**, were reported to display inhibitory activity against HIV-I reverse transcriptase with an IC_50_ of 15.5 and 12.2 μg/mL [7].

#### 2.5.3. Anti-Tumor Activity

The alisol B derivative **24** exhibited cytotoxic activity against several cancer cell lines SK-OV3 (a human ovary adenocarcinoma cell line), B16-F10 (a murine melanoma cell line), and HT1080 (a human fibrosarcoma cell line), showing ED_50_ value of 7.5, 7.5, and 4.9 μg/mL, respectively. The alisol A analogue **2**, as well as the alisol B analogues **25** and **33**, showed only weak activities in the same cell lines with ED_50_ values of 10–20 μg/mL [15]. 

In a structure-activity relationship study, twelve PT analogues were synthetically prepared from **25** and assessed for cytotoxicity against a panel of human and murine tumor cell lines. Among them, 23*S*-acetoxy-24*R*(25)-epoxy-11*β*,23*S*-dihydroxyprotost-13(17)-en-3-hydroxyimine exhibited significant cytotoxic activities against A549 (a human lung carcinoma cell line), SK-OV3, B16-F10, and HT1080 tumor cells with ED_50_ values of 10.0, 8.7, 5.2, and 3.1 μg/mL, respectively. Furthermore, 23*S*-acetoxy-13(17),24*R*(25)-diepoxy-11*β*-hydroxy-protost-3-one, 13(17),24*R*(25)-diepoxy-11*β*,23S-dihydroxyprotostan-3-one, 24*R*,25-epoxy-11*β*,23*S*-dihydroxyprotost-13(17)-en-3-one, and 11*β*,23*S*,24*R*,25-tetrahydroxyprotost-13(17)-en-3-one displayed moderate cytotoxic activities against two of these cell lines, B16-F10 and HT1080. The findings seemed to suggest that a hydroxyimino group at the C-3 position would enhance the cytotoxic activity of this class of compounds [60].

In addition, compound **25** was found to induce apoptotic cell death in human hormone-resistant prostate cancer PC-3 cells in a time- and concentration-dependent manner. The mechanism was described to be mitochondria-mediated, causing the activation of caspases-3, -8, and -9. Compound **25** was found not only to induce *Bax* (a member of the *Bcl*-2 gene family of apoptosis regulatory proteins) expression, but also to cause the translocation of *Bax* from the cytosol to the nucleus [32]. 

#### 2.5.4. Multi-Drug Resistance Reversal Activity in Cancer Therapy

The alisol B analogue **25** was suggested to have effects on reversing the multidrug resistance (MDR) of certain cancer cell lines towards standard chemo-therapy. Thus it was found to restore the sensitivity of two MDR cell lines, HepG_2_-DR and K562-DR, towards anti-tumor agents which are substrates of P-glycoprotein (P-gp) but have different modes of action. For example, **25** restored the activity of vinblastine in causing G_2_/M arrest in MDR cells. **25** increased doxorubicin accumulation in a dose dependent manner, and slowed down the efflux of rhodamin-123 from MDR cells. In addition, **25** inhibited the photoaffinity labeling of P-gp by [^125I^]iodoarylazidoprazosin and stimulated the ATPase activity of P-gp in a concentration-dependent manner. This suggested that it could be a transporter substrate for P-gp. **25** was also found to be a partial non-competitive inhibitor of P-gp when verapamil was used as a substrate [61]. 

#### 2.5.5. Anti-Complement Activity

Alisol A analogues **1**, and **2**, as well as alisol B analogues **24**, and **25**, were reported to inhibit the complement-induced hemolysis through the classical pathway [12]. **2** and **24** exhibited anti-complement activity with IC_50_ values of 130 μM and 150 μM, respectively.

11*β*,23*S*,24*R*,25-Tetrahydroxyprotost-13(17)-en-3-one, a synthetic derivatives of **24**, showed moderate inhibitory activity with an IC_50_ value of 97.1 μM. The introduction of an aldehyde group at C-23 was found to produce the most potent inhibitory effect on the complement system* in vitro* (IC_50_47.7 μM) [46].

#### 2.5.6. Other Biological Activities

The alisol B derivatives **24** and **25** were reported to exhibit muscle relaxant effects on isolated rat ileum against contractions induced by 5-isoleucine-angiotensin I, bradykinin, and acetylcholine [30]. Alisol B analogue **38** and the *seco*-PT analogue **49** showed concentration-dependent (10^−5^–10^−4^ M) inhibitory activities on the contractions induced by K^+^ in isolated aortic strips of rats [37].

Alisol A analogues **1** and **4** and the alisol B analogue **25** showed antiplasmodial activities against *Plasmodium falciparum* K1 strain with IC_50_ from 5.4 to 13.8 μM [13].

The alisol B derivatives **24**, **25**, **28**, **30**, and **33** were found to be effective in restoring choline acetyltransferase activity, and were suggested to have potential for the treatment of Alzheimer’s disease, myasthenia gravis, and gastrointestinal disorders [31].

The alisol A analogues **3**, **13**, and **14**, the aliol B analogues **39**, **42**, and **43**, the alisol E analogue **47**, and the *seco*-PT analogues **49**, **50**, and **52** were found to inhibit nitric oxide production in lipopolysaccharide-induced macrophages (IC_50_ = 8.4–68 μM). This action is suggestive of anti-inflammatory activity. In addition, the alisol A analogues **1**, **2**, and **4** and the alisol B derivatives **24**, **25**, **30**, **41**, **44**, and **46** showed potent inhibitory activity in the same bio-assay, but exhibited cytotoxic effects at concentrations above 30 μM (MTT assay). Compound **3** was found to suppress inducible nitric oxide synthase induction [11].

Compounds **2** and **24** produced a significant increase in Na^+^ excretion in saline-loaded rats when administered orally at a dose of 30 mg/kg [16]. 

Alisol B derivative **24** was reported to inhibit cell proliferation and induce apoptosis in both rat aortic smooth muscle A7r5 cells and human CEM lymphocytes. The effect was suggested to be partly due to the induction of c-Myc expression as well as the collapse of *Bax/Bcl*-2-mediated mitochondrial membrane potentials. In addition to apoptotic effect, **24** showed hypolipidemic and anti-inflammatory effects, and it was proposed to be useful for the development of drugs to prevent pathological changes associated with atherosclerosis and post-angioplasty restenosis [62].

Compounds **1**, **24**, **25**, and **33** were observed to regulate the 5-HT_3_A receptor expression in *Xenopus* oocytes. All were reported to regulate the 5-HT-induced inward peak current mediated by the human 5-HT_3_A receptor in a concentration-dependent and reversible, but non-competitive, manner with relatively low IC_50_ values (1.7–3.5 μM) [14].

Compound **3** showed inhibitory activity to alpha-glucosidase in a dose-dependent manner (0.125–2.5 mM). Since the total aqueous ethanol extract of Alismatis Rhizoma (25 μg/mL) could inhibit alpha-glucosidase activity by 34.06%, comparing to acarbose (0.5 mM) by 47.08% [63], the PTs present in the plant are likely involved in the inhibitory process.

## 3. Fusidane Triterpenes

**Figure 8:**
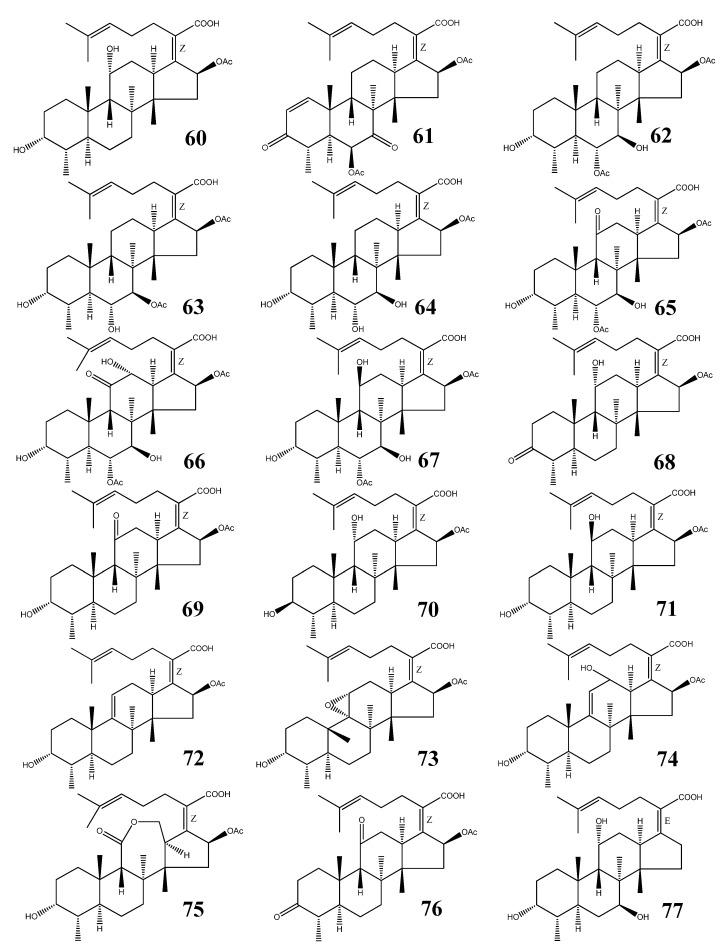
Structures of fusidane triterpenes.

Fusidane triterpenes (FT) belong to a small group of 29-*nor* protostane triterpenes (Figure 8). They are antimicrobial agents produced by fungal species (Table 2) [64,65,66,67,68,69,70,71,72,73,74,75,76,77,78,79,80,81,82,83,84,85,86,87]. Though only a few structures of this class have been reported to date, FTs play important roles as antibiotic agents. The most important representative is fusidic acid (**60**).

**Table 2:** Naturally occurring fusidane triterpens.

No.	Name	M.F.	Source	References
**60**	Fusidic acid	C_31_H_48_O_6_	*Acremonium fusidioides*, *Calcarisporium arbuscula*, *Cephalosporium lamellaecula*, *C. acremonium, Epidermophyton floccosum*, *Fusidium coccineum*, *Gabarnaudia tholispora, Mucor ramannianus*, *Paecilomyces fusidioides*	[64,65,66,67]
**61**	Helvolic acid	C_33_H_44_O_8_	*Aspergillus fumigatus*, *A. sydowi*, *A.* sp. CY725, *Alternaria *sp. FL25, *Cephalosporium caerulens*, *Emericellopsis terricola*, *Metarhizium anisopliae*, *Penicilliopsis* sp., *Pichia guilliermondii*, *Sarocladium oryzae*	[1,66,75,79]
**62**	Cephalosporin P1	C_33_H_50_O_8_	*Cephalosporium acremonium*, *Cladosporium* sp.	[66,81,84,85,86]
**63**	Isocephalosporin P1	C_33_H_50_O_8_	*Cephalosporium acremonium*	[84]
**64**	Monodesacetyl cephalosporin P1	C_3__1_H_48_O_7_	*Cephalosporium acremonium*	[84]
**65**	Viridominic acid A	C_33_H_48_O_9_	*Cladosporium* sp.	[66,85,86]
**66**	Viridominic acid B	C_33_H_48_O_10_	*Cladosporium* sp.	[66,85,86]
**67**	Viridominic acid C	C_33_H_50_O_9_	*Cladosporium* sp.	[66,85,86]
**68**	3-Ketofusidic acid	C_31_H_46_O_6_	*Epidermophyton floccosum*, *Fusidium coccineum*	[65,67]
**69**	11-Ketofusidic acid	C_31_H_46_O_6_	*Fusidium coccineum*	[65]
**70**	3-Epifusidic acid	C_31_H_48_O_6_	*Fusidium coccineum*	[65]
**71**	11-Epifusidic acid	C_31_H_48_O_6_	*Fusidium coccineum*	[65]
**72**	9,11-Anhydrofusidic acid	C_31_H_46_O_5_	*Fusidium coccineum*	[65]
**73**	9,11-Anhydro-9 *α*,11*α*-epoxyfusidic acid	C_31_H_46_O_6_	*Fusidium coccineum*	[65]
**74**	9,11-Anhydro-12-hydroxyfusidic acid	C_31_H_46_O_6_	*Fusidium coccineum*	[65]
**75**	Fusilactidic acid	C_31_H_46_O_7_	*Fusidium coccineum*	[65]
**76**	3,11-Diketofusidic acid	C_31_H_44_O_6_	*Epidermophyton floccosum*	[67]
**77**	16-Deacetoxy-7 *β*-hydroxy fusidic acid	C_29_H_46_O_5_	*Acremonium crotocinigenum*	[87]

### 3.1. Structural Features

All FTs contain double-bonds at the 17(20) and 24(25) positions, a carboxylic acid group (C-21), and a 16-OAc, with exception of 16-deacetoxy-7-*β*-hydroxy fusidic acid (**77**). Oxygen substituents can be present at positions C-6, C-7, C-11, and C-12. Fusilactidic acid (**75**) is a unique FT bearing a seven-membered ring. 

### 3.2. Fusidic Acid

Fusidic acid (**60**) was first isolated from *Fusidium coccineum* by Godtfredsen [64] and also reported from several other fungal sources (Table 2) [65,66,67]. It has been clinically used as an antibiotic since 1962 in both systemic and topical therapies for staphylococcal infections [68]. It exhibits potent effects against staphylococci, including the methicillin-resistant *Staphylococcus aureus* (MRSA) and the coagulase-negative staphylococcal species. 

Fusidic acid distributes well in various tissue, exhibits low toxicity and allergic reactions; and it has little cross-resistance with other clinically used antibiotics. Though never approved for use in the United States, fusidic acid is marketed in more than twenty countries with 21 million annual prescriptions [69]. The global problem of microbial resistance has now led to a renewed interest in its use. Since 2006, this “old” antibiotic has received attention in the United States mainly because no recommended oral antibiotics (such as oxacillin, cloxacillin, dicloxacillin, and cephalexin) have shown useful activity against MRSA. To date, phase 2 clinical trials has finished and the results supported proceeding to phase 3 studies [70]. 

Fusidic acid (**60**) acts as a protein synthesis inhibitor, binding to elongation factor G (EF-G). The binding site was identified to be a pocket between domains I, II, and III of EF-G (EF-G consists of 5 domains). This binding results in a conformational intermediate structure between the GDP- and GTP-bound forms [71]. Due to its unique action mechanism, **60** has shown no cross-resistance with any other class of antibiotic. 

The structure-activity relationship of fusidic acid (**60**) and related compounds have been extensively studied. The tetracyclic fusidane skeleton, lipophilic side-chain, and the carboxylic acid group at C-20 seem to be essential for its biological activity. The orientation of the lipophilic side-chain, rather than the double bond, is crucial to the antibacterial activity [72].

Structural modifications of **60** have shown that, among 51 derivatives, none displayed antibacterial activity better than the parent compound, and only one derivative, 24,25-dihydrofusidic acid, turned out to be as active as **60** itself [73].

### 3.3. Other Fusidane Triterpenes

Helvolic acid (**61**) was isolated from *Aspergillus fumigatus* during World War II. It showed bacteriostatic activity against gram-positive organisms, but had no effect against gram-negatives [74,75]. Subsequently, it showed significant antimicrobial activity against a wide range of microorganisms including fungi [76,77,78,79]. Compound **61** also exhibited synergistic effects with erythromycin on all tested multi-drug resistant *Staphylococcus aureus* and with penicillin and tetracycline on some multi-drug resistant *S. aureus* strains. Enhanced effect was also found in time-kill studies on multi-drug resistant *S. aureus* strains [80].

Cephalosporin P1 (**62**) was discovered from the culture fluid of *Cephalosporium acremonium* in 1951, which exhibited potent activity against methicillin-sensitive, methicillin-resistant, and vancomycin-intermediate *Staphylococcus aureus* [81,82,83]. The complete cross-resistance between **60** and **62** was reported, but the nature and location of *fus*A (the gene that encodes EF-G) mutations selected by these two agents in *S. aureus* appeared to be different. The interaction of them with EF-G may also differ based on the examination of their effects on translocation and peptide bond formation using cell-free assays [83].

Isocephalosporin P1(**63**), and monodesacetyl cephalosporin P1(**64**), together with **62**, were isolated from an organic extract of the fermentation broth of *Cephalosporium acremonium* [84]. Viridominic acids A (**65**), B (**66**), and C (**67**), together with **62, **were obtained from the culture filtrate of a *Cladosporium* sp. and reported to possess chlorosis-inducing activity against higher plant [85,86].

3-Ketofusidic acid (**68**), 11-ketofusidic acid (**69**), 3-epifusidic acid (**70**), 11-epifusidic acid (**71**), 9,11-anhydrofusidic acid (**72**), 9,11-anhydro-9*α*,11*α*-epoxyfusidic acid (**73**), 9,11-anhydro-12-hydroxyfusidic acid (**74**), and fusilactidic acid (**75**) were all isolated from the industrial fermentation of *Fusidium coccineum* [65]. They showed antibiotic activity against gram-positive bacteria, in particular, a strain of *Staphylococcus aureus*, but they had no activity against the gram-negative bacterium *Escherichia coli* [66].

3,11-Diketofusidic acid (**76**), together with **60**, was isolated from* Epidermophyton floccosum* in 1983 [67]. From a fermentation of the mitosporic fungus *Acremonium crotocinigenum*, 16-deacetoxy-7*β*-hydroxy fusidic acid was isolated (**77**). They were all less potent than **60** against a battery of multidrug-resistant and methicillin-resistant *Staphylococcus aureus* (MRSA) stains [87].

### 3.4. Biosynthesis of Fusidane Triterpenes

FTs share a similar biosynthetic pathway with PTs leading to the formation of protosteryl C-20 cation. 3*β*-Hydroxy-protosta-17(20)*Z*, 24-diene is supposed to be the precursor of FTs. After the formation of 3*β*-hydroxy-protosta-17(20)*Z*, 24-diene, further demethylation process of C-29 is catalyzed to produce the FT skeleton. A novel oxidosqualene cyclase (OSC), namely oxidosqualene: protostadienol cyclase (OSPC), produced in *Aspergillus fumigatus* was reported to be involved in the biosynthesis of helvolic acid [88,89]. The stabilization of the C-20 protosteryl cation by the active site Phe701 of OSPC through cation-π interactions is important for the product outcome of protostadienol [90]. Three genes (AfuOSC3, AfuSDR1, and CYP5081A1) have been characterized in the early steps of helvolic acid biosynthesis. AfuOSC3 is responsible for the formation of the basic carbon skeleton 3*β*-hydroxy-protosta-17(20)*Z*, 24-diene, whereas both AfuSDR1 and CYP5081A1 presumably work together to catalyze the demethylation of C-29 [89].

## 4. Conclusions and Future Prospects

PTs represent a compound class with a unique triterpene structure and they have been found to exhibit diverse biological activities in a broad range of* in vitro* and* in vivo* studies. To date only 59 PTs have been reported, with the majority isolated from the genus *Alisma*, mainly *A. orientale* and *A.* *plantago-aquatica*. Further phytochemical investigations on other *Alisma* plant species are warranted. 

Details of the PT biosynthetic pathway are lacking at this time. It is likely that the *Alisma* genus possesses unique and specific enzyme systems capable of catalyzing the complex biosynthetic pathway, making the PT biosynthetic pathway is worthy of further studies in the future. 

Because most reported PTs have not been investigated conclusively for their biological activities, and because some derivatives have shown significant effects in the bioassay systems, further biological studies on these compounds are anticipated to reveal interesting results, especially with respect to lipotropism, liver protection, and anti-hepatitis B activity. A better understanding of their biological activities would shed light on the rational use of Alismatis Rhizoma.

Fusidic acid, after decades of clinical use, remains a promising antibiotic agent due to its potency against MRSA, low degree of toxicity and allergic reactions, and no cross-resistance with other clinically used antibiotics. A new dosing regimen of fusidic acid has been developed in the United States in order to minimize the fusidic acid resistance selection and obviate the negative effects of protein binding [91]. This new strategy warrants further development of this antimicrobial agent. 

## References

[1] Hattori T., Igarashi H., Iwasaki S., Okuda S. (1969). Helvolic acid and related compounds. VI. Isolation of 3*β*-hydroxy-4*β*-methylfusida-17(20)[16,21-*cis*],24-diene (3*β*-hydroxyprotosta-17(20)[16,21-*cis*],24-diene) and a related triterpene alcohol. Tetrahedron Lett..

[2] Awaad A.S., Singh V.K., Govil J.N. (2010). Recent Progress in Medicinal Plants.

[3] Murata T., Imai Y., Hirata T., Miyamoto M. (1970). Biological-active triterpenes of *Alismatis rhizoma*. I. Isolation of the alisols. Chem. Pharm. Bull..

[4] Kimura H., Ogata T., Sato Y. (1992). Preparation of 16-ketoalisol A, and alisol A derivatives for treatment of liver disorders.

[5] Jiang Z.Y., Zhang X.M., Zhang F.X., Liu N., Zhao F., Zhou J., Chen J.J. (2006). A new triterpene and anti-hepatitis B virus active compounds from *Alisma orientalis*. Planta Med..

[6] Wang X.B., Kong L.Y. (2007). Alisol F 24-acetate: (24*R*)-24-acetoxy-11*β*,25-dihydroxy-16*β*,23*β*-epoxyprotost-13(17)-en-3-one. Acta Crystallogr. E.

[7] Rukachaisirikul V., Pailee P., Hiranrat A., Tuchinda P., Yoosook C., Kasisit J., Taylor W.C., Reutrakul V. (2003). Anti-HIV-1 protostane triterpenes and digeranylbenzophenone from trunk bark and stems of *Garcinia speciosa*. Planta Med..

[8] Miyaichi Y., Segawa A., Tomimori T. (2006). Studies on nepalese crude drugs. Chemical constituents of dronapuspi, the whole herb of *Leucas cephalotes* SPRENG. Chem. Pharm. Bull..

[9] Murata T., Shinohara M., Hirata T., Kamiya K., Nishikawa M., Miyamoto M. (1968). New triterpenes of *Alismaplantago-aquatica* var. *orientale*. Tetrahedron Lett..

[10] Chau V.M., Phan V.K., Pham H.Y., Tran T.H., Braca A. (2007). Protostane-type triterpenes from the rhizomes of *Alisma plantago-aquatica*. Tap Chi Hoa Hoc.

[11] Matsuda H., Kageura T., Toguchida I., Murakami T., Kishi A., Yoshikawa M. (1999). Effects of sesquiterpenes and triterpenes from the rhizome of *Alisma orientale* on nitric oxide production in lipopolysaccharide-activated macrophages: Absolute stereostructures of alismaketones-B 23-acetate and -C 23-acetate. Bioorg. Med. Chem. Lett..

[12] Matsuda H., Tomohiro N., Yoshikawa M., Kubo M. (1998). Studies on Alismatis Rhizoma. II. Anti-complementary activities of methanol extract and terpene components from Alismatis Rhizoma (Dried rhizome of *Alisma orientale*). Biol. Pharm. Bull..

[13] Adams M., Gschwind S., Zimmermann S., Kaiser M., Hamburger M. (2011). Renaissance remedies: Antiplasmodial protostane triterpenoids from *Alisma plantago-aquatica* L. (Alismataceae). J. Ethnopharmacol..

[14] Lee J.H., Lee Y.J., Kang S.W., Kim Y., Shin M., Hong M., Seo E.K., Kim S.H., Nah S.Y., Bae H. (2010). Effects of protostane-type triterpenoids on the 5-HT_3A_ receptor-mediated ion current in *Xenopus oocytes*. Brain Res..

[15] Lee S., Kho Y., Min B., Kim J., Na M., Kang S., Maeng H., Bae K. (2001). Cytotoxic triterpenoids from *Alismatis Rhizoma*. Arch. Pharmacal Res..

[16] Hikino H., Iwakawa T., Oshima Y., Nishikawa K., Murata T. (1982). Efficacy of oriental drugs. 34. Diuretic principles of *Alisma plantago-aquatica* var. *orientale* rhizomes. Shoyakugaku Zasshi.

[17] Murata T., Shinohara M., Hirata T., Miyamoto M. (1968). Structures of alisol B and alisol A monacetate-occurrence of a facile acyl migration. Tetrahedron Lett..

[18] Wo L., Luo G., Wang B., Zhu W. (2005). Studies on triterpenes chemical constituents in rhizome of *Alisma gramineum*. Zhongguo Zhongyao Zazhi.

[19] Yoshikawa M., Hatakeyama S., Tanaka N., Fukuda Y., Yamahara J., Murakami N. (1993). Crude drugs from aquatic plants. I. On the constituents of Alismatis Rhizoma. (1). Absolute stereostructures of alisols E 23-acetate, F, and G, three new protostane-type triterpenes from Chinese *Alismatis Rhizoma*. Chem. Pharm. Bull..

[20] Nakajima Y., Satoh Y., Katsumata M., Tsujiyama K., Ida Y., Shoji J. (1994). Terpenoids of *Alisma orientale* rhizomes and the crude drug *Alismatis rhizoma*. Phytochemistry.

[21] Kato T., Tomita M., Takigawa M., Iwasaki H., Hirukawa T., Yamahara J. (1994). Inhibitory effects and active constituents of *Alisma rhizomes* on vascular contraction induced by high concentration of KCl. Bull. Chem. Soc. Jpn..

[22] Zeng L., Pen X., Zhang R.Y. (1995). Alizexol A, a novel protostane type of triterpene from *Alisma orientalis* (SAM) Juzep. Chin. Chem. Lett..

[23] Yoshikawa M., Tomohiro N., Murakami T., Ikebata A., Matsuda H., Matsuda H., Kubo M.  (1999). Studies on alismatis rhizoma. III. Stereostructures of new protostane-type triterpenes, alisols H, I, J-23-acetate, K-23-acetate, L-23-acetate, M-23-acetate, and N-23-acetate, from the dried rhizome of *Alisma orientale*. Chem. Pharm. Bull..

[24] Peng G.P., Zhu G.Y., Lou F.C. (2002). Two novel terpenoids from *Alisma orientalis* Juzep. Tianran Chanwu Yanjiu Yu Kaifa.

[25] Peng G.P., Zhu G.Y., Lou F.C. (2002). Terpenoids from *Alisma orientalis* Juzep. Tianran Chanwu Yanjiu Yu Kaifa.

[26] Zhou A.C., Zhang C.F., Zhang M. (2008). A new protostane triterpenoid from the rhizome of *Alisma orientale*. Zhongguo Tianran Yaowu.

[27] Hu X.Y., Guo Y.Q., Gao W.Y., Zhang T.J., Chen H.X. (2008). Two new triterpenes from the rhizomes of *Alisma orientalis*. J. Asian Nat. Prod. Res..

[28] Hu X.Y., Guo Y.Q., Gao W.Y., Chen H.X., Zhang T.J. (2008). A new triterpenoid from *Alisma orientalis*. Chin. Chem. Lett..

[29] Xu N., Zhang H., Xie X. (2012). A new triterpene in rhizome of *Alisma orientale*. Zhongcaoyao.

[30] Yun H.S., Chung S.H., Kim Y.S. (1981). Effect of alisols isolated from *Alisma orientale* Jazep. against several agonists in isolated rat ileum. Soul Taehakkyo Saengyak Yonguso Opjukjip.

[31] Yamada S., Yamaguchi T., Naito T., Hashimoto K. (1995). Alisol B analogs for restoration of choline acetyltransferase.

[32] Huang Y.T., Huang D.M., Chueh S.C., Teng C.M., Guh J.H. (2006). Alisol B acetate, a triterpene from Alismatis rhizoma, induces Bax nuclear translocation and apoptosis in human hormone-resistant prostate cancer PC-3 cell. Cancer Lett..

[33] Chang I.M., Kim Y.S., Yun H.S., Kim S.O. (1982). Liver-protective activities of alisol compounds against carbon tetrachloride intoxication. Saengyak Hakhoe Chi (Hanguk Saengyak Hakhoe).

[34] Fukuyama Y., Geng P., Wang R., Yamada T., Nakagawa K. (1988). 11-Deoxyalisol C and alisol D: New protostane-type triterpenoids from *Alisma plantago-aquatica*. Planta Med..

[35] Geng P., Fukuyama Y., Yamada T., Rei W., Jinxian B., Nakagawa K. (1988). Triterpenoids from the rhizome of *Alisma plantago-aquatica*. Phytochemistry.

[36] Yoshikawa M., Hatakeyama S., Tanaka N., Matsuoka T., Yamahara J., Murakami N. (1993). Crude drugs from aquatic plants. II. On the constituents of the rhizome of *Alisma orientale* Juzep. originating from Japan, Taiwan, and China. Absolute stereostructures of 11-deoxyalisols B and B 23-acetate. Chem. Pharm. Bull..

[37] Yoshikawa M., Murakami T., Ikebata A., Ishikado A., Murakami N., Yamahara J., Matsuda H. (1997). Absolute stereostructures of alismalactone 23-acetate and alismaketone-a 23-acetate, new seco-protostane and protostane-type triterpenes with vasorelaxant effects from Chinese *Alismatis Rhizoma*. Chem. Pharm. Bull..

[38] Jin H.-G., Jin Q., Ryun K.A., Choi H., Lee J.H., Kim Y.S., Lee D.G., Woo E.-R. (2012). A new triterpenoid from *Alisma orientale* and their antibacterial effect. Arch. Pharmacal Res..

[39] Zhao M., Xu L.J., Che C.T. (2007). Alisolide, alisols O and P from the rhizome of *Alisma orientale*. Phytochemistry.

[40] Liu X., Li S.L., Zhou Y., Song J.Z., Zheng Y.F., Peng G.P., Xu H.X. (2010). Characterization of protostane triterpenoids in *Alisma orientalis* by ultra-performance liquid chromatography coupled with quadrupole time-of-flight mass spectrometry. Rapid Commun. Mass Spectrom..

[41] Kamiya K., Murata T., Nishikawa M. (1970). Biological-active triterpenes of Alismatis rhizoma. III. X-ray crystallography of alisol A (23, 24)-acetonide 11-monobromoacetate. Chem. Pharm. Bull..

[42] Peng G.P., Lou F.C. (2001). Chemical studies on *Alisma orientalis* Juzep. Tianran Chanwu Yanjiu Yu Kaifa.

[43] Murakami N., Yagi N., Murakami T., Yoshikawa M. (1996). Electrochemical transformation of protostane type triterpenes. Chem. Pharm. Bull..

[44] Ye Y.P., Sun C.R., Li X.Y., Sun H.X., Pan Y.J. (2003). Alisol B monoacetate from the rhizome of *Alisma orientale*. Acta Crystallogr. E.

[45] Yamaguchi K., Ida Y., Nakajima Y., Satoh Y., Shoji J. (1994). Absolute stereostructure of 13,17-epoxyalisol B 23-acetate isolated from *Alisma orientale*. Acta Crystallogr. C.

[46] Lee S.M., Kim J.H., Zhang Y., An R.B., Min B.S., Joung H., Lee H.K. (2003). Anti-complementary activity of protostane-type triterpenes from *Alismatis rhizoma*. Arch. Pharm. Res..

[47] Xu R., Fazio G.C., Matsuda S.P.T. (2004). On the origins of triterpenoid skeletal diversity. Phytochemistry.

[48] Corey E.J., Virgil S.C., Cheng H., Baker C.H., Matsuda S.P.T., Singh V., Sarshar S. (1995). New insights regarding the cyclization pathway for sterol biosynthesis from (*S*)-2,3-oxidosqualene. J. Am. Chem. Soc..

[49] Corey E.J., Cheng H. (1996). Conversion of a C20 2,3-oxidosqualene analog to tricyclic structures with a five-membered C-ring by lanosterol synthase. Further evidence for a C-ring expansion step in sterol biosynthesis. Tetrahedron Lett..

[50] Hess B.A. (2002). Concomitant C-ring expansion and D-ring formation in lanosterol biosynthesis from squalene without violation of Markovnikov’s Rule. J. Am. Chem. Soc..

[51] Hess B.A. (2003). Formation of the C ring in the lanosterol biosynthesis from squalene. Org. Lett..

[52] Abe I. (2007). Enzymatic synthesis of cyclic triterpenes. Nat. Prod. Rep..

[53] Zheng Y.F., Zhu Y.L., Peng G.P. (2006). Transformation of alisol B 23-acetate in processing of *Alisma orientalis*. Zhongcaoyao.

[54] Yoshikawa M., Yamaguchi S., Chatani N., Nishino Y., Matsuoka T., Yamahara J., Murakami N., Matsuda H., Kubo M. (1994). Crude drugs from aquatic plants. III. Quantitative analysis of triterpene constituents in alismatis rhizoma by means of high-performance liquid chromatography on the chemical change of the constituents during alismatis rhizoma processing. Yakugaku Zasshi.

[55] Brown A.J. (2009). 24(*S*),25-Epoxycholesterol: A messenger for cholesterol homeostasis. Int. J. Biochem. Cell Biol..

[56] Spencer T.A. (1994). The squalene dioxide pathway of steroid biosynthesis. Acc. Chem. Res..

[57] Zhang Q., Jiang Z.Y., Luo J., Cheng P., Ma Y.B., Zhang X.M., Zhang F.X., Zhou J., Chen J.J.  (2008). Anti-HBV agents. Part 1: Synthesis of alisol A derivatives: A new class of hepatitis B virus inhibitors. Bioorg. Med. Chem. Lett..

[58] Zhang Q., Jiang Z.Y., Luo J., Liu J.F., Ma Y.B., Guo R.H., Zhang X.M., Zhou J., Chen J.J. (2009). Anti-HBV agents. Part 2: Synthesis and *in vitro* anti-hepatitis B virus activities of alisol A derivatives. Bioorg. Med. Chem. Lett..

[59] Zhang Q., Jiang Z.Y., Luo J., Ma Y.B., Liu J.F., Guo R.H., Zhang X.M., Zhou J., Niu W., Du F.F. (2009). Anti-HBV agents. Part 3: Preliminary structure-activity relationships of tetra-acylalisol A derivatives as potent hepatitis B virus inhibitors. Bioorg. Med. Chem. Lett..

[60] Lee S., Min B., Bae K. (2002). Chemical modification of alisol B 23-acetate and their cytotoxic activity. Arch. Pharm. Res..

[61] Wang C., Zhang J.X., Shen X.L., Wan C.K., Tse A.K.W., Fong W.F. (2004). Reversal of P-glycoprotein-mediated multidrug resistance by Alisol B 23-acetate. Biochem. Pharmacol..

[62] Chen H.W., Hsu M.J., Chien C.T., Huang H.C. (2001). Effect of alisol B acetate, a plant triterpene, on apoptosis in vascular smooth muscle cells and lymphocyte. Eur. J. Pharmacol..

[63] Li Q., Qu H. (2012). Study on the hypoglycemic activities and metabolism of alcohol extract of *Alismatis Rhizoma*. Fitoterapia.

[64] Godtfredsen W.O., Jahnsen S., Lorck H., Roholt K., Tybring L. (1962). Fusidic acid, a new antibiotic. Nature.

[65] Godtfredsen W.O., Rastrup-Andersen N., Vangedal S., Ollis W.D. (1979). Metabolites of *Fusidium coccineum*. Tetrahedron.

[66] Cole R.J., Schweikert M.A. (2003). Handbook of Secondary Fungal Metabolites, Volume 1.

[67] Perry M.J., Hendricks-Gittins A., Stacey L.M., Adlard M.W., Noble W.C. (1983). Fusidane antibiotics produced by dermatophytes. J. Antibiot..

[68] Jones R.N., Mendes R.E., Sader H.S., Castanheira M. (2011). *In vitro* antimicrobial findin–2009) gram-positive organisms collected in the United States. Clin. Infect. Dis..

[69] Kraus C.N., Burnstead B.W. (2011). The safety record of fusidic acid in non-US markets: A focus on skin infections. Clin. Infect. Dis..

[70] Craft J.C., Moriarty S.R., Clark K., Scott D., Degenhardt T.P., Still J.G., Corey G.R., Das A., Fernandes P. (2011). A randomized, double-blind phase 2 study comparing the efficacy and safety of an oral fusidic acid loading-dose regimen to oral linezolid for the treatment of acute bacterial skin and skin structure infections. Clin. Infect. Dis..

[71] Farrell D.J., Castanheira M., Chopra I. (2011). Characterization of global patterns and the genetics of fusidic acid resistance. Clin. Infect. Dis..

[72] Duvold T., Sorensen M.D., Bjoerkling F., Henriksen A.S., Rastrup-Andersen N. (2001). Synthesis and conformational analysis of fusidic acid side chain derivatives in relation to antibacterial activity. J. Med. Chem..

[73] Godtfredsen W.O., Von D.W., Tybring L., Vangedal S. (1966). Fusidic acid derivatives. I. Relation between structure and antibacterial activity. J. Med. Chem..

[74] Waksman S.A., Horning E.S., Spencer E.L. (1942). Production of two antibacterial substances, fumigacin and clavacin. Science.

[75] Chain E., Florey H.W., Jennings M.A., Williams T.I. (1943). Helvolic acid, an antibiotic produced by *Aspergillus fumigatus*, mut. helvola Yuill. Br. J. Exp. Pathol..

[76] Li X.J., Zhang Q., Zhang A.L., Gao J.M. (2012). Metabolites from *Aspergillus fumigatus*, an endophytic fungus associated with *Melia azedarach*, and their antifungal, antifeedant, and toxic activities. J. Agric. Food Chem..

[77] Jennings M.A. (1945). Activity of helvolic acid against *Mycobacterium tuberculosis*. Nature.

[78] Feng C., Ma Y. (2010). Isolation and anti-phytopathogenic activity of secondary metabolites from *Alternaria* sp. FL25, an endophytic fungus in *Ficus carica*. Yingyong Yu Huanjing Shengwu Xuebao.

[79] Zhao J., Mou Y., Shan T., Li Y., Zhou L., Wang M., Wang J. (2010). Antimicrobial metabolites from the endophytic fungus *Pichia guilliermondii* isolated from *Paris polyphylla* var. *yunnanensis*. Molecules.

[80] Qin L., Li B., Guan J., Zhang G. (2009). *In vitro* synergistic antibacterial activities of helvolic acid on multi-drug resistant *Staphylococcus aureus*. Nat. Prod. Res..

[81] Burton H.S., Abraham E.P. (1951). Isolation of antibiotics from a species of *Cephalosporium*. Cephalosporins P_1_, P_2_, P_3_, P_4_, and P_5_. Biochem. J..

[82] Ritchie A.C., Smith N., Florey H.W. (1951). Some biological properties of cephalosporin P1. Br. J. Pharmacol. Chemother..

[83] O’Neill A.J., Bostock J.M., Morais M.A., Chopra I. (2002). Antimicrobial activity and mechanisms of resistance to cephalosporin P1, an antibiotic related to fusidic acid. J. Antimicrob. Chemother..

[84] Chou T.S., Eisenbraun E.J., Rapala R.T. (1969). Chemistry of steroid acids from *Cephalosporium acremonium*. Tetrahedron.

[85] Kaise H., Ogawa Y., Sassa T., Munakata K. (1970). Chlorosis-inducing substances produced by a fungus. I. Isolation and biological activities of viridominic acids A, B, C, and cephalosporin P1. Agric. Biol. Chem..

[86] Kaise H., Munakata K., Sassa T. (1972). Structures of viridominic acids A and B, new chlorosis-inducing metabolites of a fungus. Tetrahedron Lett..

[87] Evans L., Hedger J.N., Brayford D., Stavri M., Smith E., O’Donnell G., Gray A.I., Griffith G.W., Gibbons S. (2006). An antibacterial hydroxy fusidic acid analogue from *Acremonium crotocinigenum*. Phytochemistry.

[88] Lodeiro S., Xiong Q., Wilson W.K., Ivanova Y., Smith M.L., May G.S., Matsuda S.P.T. (2009). Protostadienol biosynthesis and metabolism in the pathogenic fungus *Aspergillus fumigatus*. Org. Lett..

[89] Mitsuguchi H., Seshime Y., Fujii I., Shibuya M., Ebizuka Y., Kushiro T. (2009). Biosynthesis of steroidal antibiotic fusidanes: functional analysis of oxidosqualene cyclase and subsequent tailoring enzymes from *Aspergillus fumigatus*. J. Am. Chem. Soc..

[90] Kimura M., Kushiro T., Shibuya M., Ebizuka Y., Abe I. (2010). Protostadienol synthase from *Aspergillus fumigatus*: Functional conversion into lanosterol synthase. Biochem. Biophys. Res. Commun..

[91] Fernandes P., Pereira D. (2011). Efforts to support the development of fusidic acid in the United States. Clin. Infect. Dis..

